# Characterization of the complete chloroplast genome of *Melampyrum roseum*

**DOI:** 10.1080/23802359.2019.1670747

**Published:** 2019-09-27

**Authors:** Yong-Chao Li, Xiu-Ren Zhou, Jing Yang, Qing-Yun Fu

**Affiliations:** School of Life Science and Technology, Henan Institute of Science and Technology, Xinxiang, Henan Province, China

**Keywords:** *Melampyrum roseum*, chloroplast genome, phylogenetic analysis

## Abstract

In this study, the complete chloroplast (cp) genome of *Melampyrum roseum* was determined through Illumina sequencing method. The complete cp genome of *M. roseum* was 143,896 bp in length and contained a pair of IR regions (25,210 bp) separated by a small single-copy region (10,292 bp) and a large single-copy region (83,184 bp). The cp genome of *M. roseum* encoded 117 genes including 78 protein-coding genes, 31 tRNA genes, and 8 rRNA genes. The overall GC content of *M. roseum* cp genome is 38.1%. By phylogenetic analysis using maximum-likelihood (ML) method, *M. roseum* was placed in Rhinantheae clade as expected.

*Melampyrum roseum*, belonging to Rhinantheae (Orobanchaceae) is an annual entomophilous plant, which forms several raceme-like inflorescences with more than eight flowers, which bloom sequentially. In this study, we finished the chloroplast (cp) genome of *M. roseum* using next-generation sequencing, aiming to figure out its phylogenetic position precisely.

Plant materials of *M. roseum*, sequenced in this study, were collected from the bank region of Heili River, Ningchen in inner Mongolia (41°37'N, 118°80'E). Both plant materials and total genomic DNA, that were extracted from fresh young leaves using cetyltrimethylammonium bromide (CTAB) method, were stored in Henan Institute of Science and Technology. The corresponding specimen was also stored in herbarium of Henan Institute of Science and Technology (accession no. IST-20181004-MR).

For high-throughput sequencing (NGS), paired-end library from DNA extracts was prepared with a NEBNext Library building kit, following manufacturer’s protocol. Then, the library was sequenced on an Illumina HiSeq2500 platform (Illumina, San Diego, CA). After reads quality filtration, the clean reads were assembled by SPAdes 3.11.0 (Bankevich et al. 2012). We used cp genome of *Schwalbea americana* (accession no. HG738866) as a reference sequence to align the contigs and identify gaps. To fill the gap, Price (Ruby et al. 2013) and MITObim v1.8 (Hahn et al. 2013) were applied and Bandage (Wick et al. 2015) was used to identify the borders of the IR, LSC, and SSC regions. The complete sequence was primarily annotated by Plann (Huang et al. 2015) combined with manual correction. All tRNAs were confirmed using the tRNAscan-SE search server (Lowe and Eddy 1997). Other protein-coding genes were verified by BLAST search on the NCBI website (http://blast.ncbi.nlm.nih.gov/), and manual correction for start and stop codons were conducted. This complete cp genome sequence together with gene annotations were submitted to GenBank under the accession number MN075942.

The cp genome of *M. roseum* is a typical quadripartite structure with a length of 143,896 bp. The whole cp genome contains a large single-copy (LSC) region of 85,585 bp, a small single-copy (SSC) region of 10,292 bp, and two inverted repeat (IRs) regions of 25,210 bp each. The cp genome possesses 117 genes, including 78 protein-coding genes, 8 rRNA genes (4 rRNA species), and 31 tRNA genes. The overall GC content of the cp genome is 38.1%.

For phylogenetic analysis assessing the relationship of this plastid, we selected other 31 lamiids cp genomes to construct a genome-wide alignment. We took plastids of the Gentianales plant as the outgroup. The genome-wide alignment of all cp genomes was done by HomBlocks (Bi et al. 2018), resulting in 79,500 positions in total. The whole-genome alignment was analyzed by IQ-TREE version 1.6.6 (Nguyen et al. 2015) under the TVM + F + R4 model. The tree topology was verified under both 1000 bootstrap and 1000 replicates of SH-aLRT test. As shown in Figure 1, the phylogenetic positions of these 32 cp genomes were successfully resolved with full bootstrap supports across almost all nodes. *Melampyrum roseum* was placed in Orobanchaceae clade and exhibited as sister branch compared to other Rhinantheae species.

**Figure 1.**
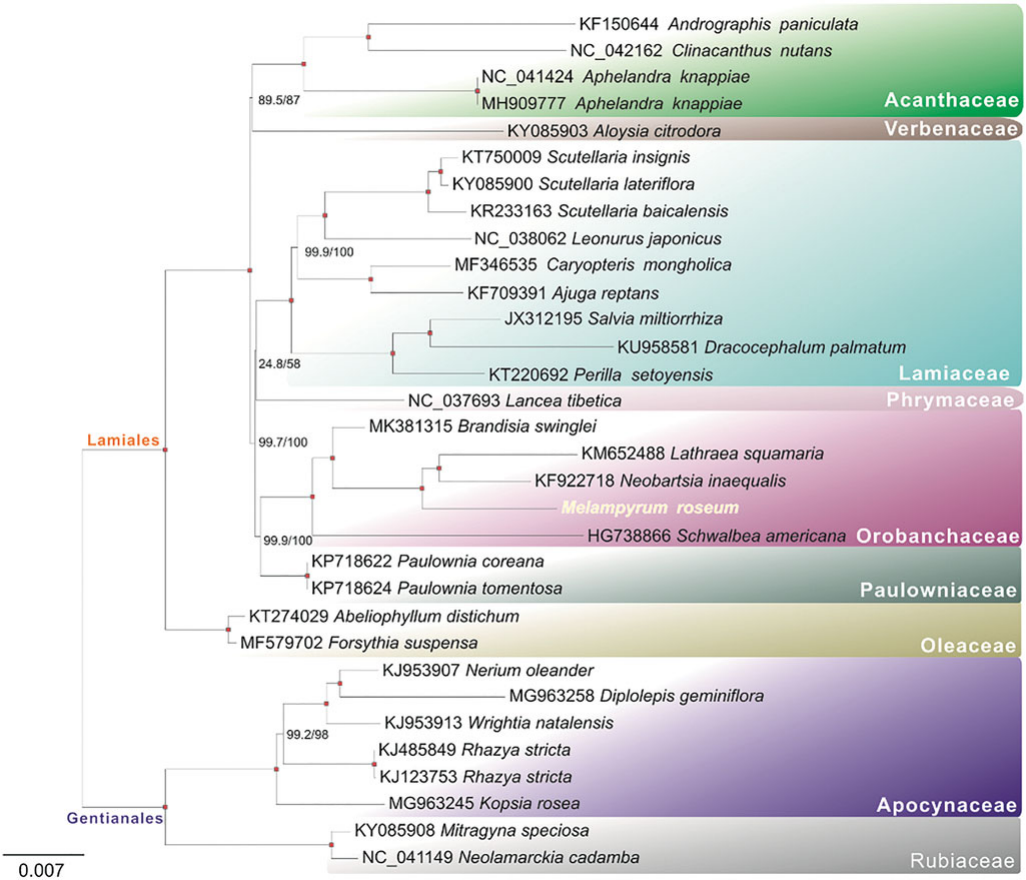
Phylogenetic tree yielded by ML analysis of 32 lamiids cp genomes. Nodes harvesting both full bootstrap and SH-like aLRT values are indicated by pink points. Scale indicates substitutions per site.
